# Migration and Histologic Effects of Visible Implant Elastomer (VIE) and Passive Integrated Transponder (PIT) Tags in the Marine Toad (*Rhinella marina*)

**DOI:** 10.3390/ani11113255

**Published:** 2021-11-14

**Authors:** Megan L. Cabot, Brigid V. Troan, Kimberly Ange-van Heugten, Rodney W. Schnellbacher, Dustin Smith, Frank Ridgley, Larry J. Minter

**Affiliations:** 1 Department of Clinical Sciences, North Carolina State University, Raleigh, NC 27606, USA; Brigid.Troan@nczoo.org (B.V.T.); Jb.Minter@nczoo.org (L.J.M.); 2 North Carolina Zoo, Asheboro, NC 27205, USA; Dustin.Smith@nczoo.org; 3 Department of Animal Science, North Carolina State University, Raleigh, NC 27607, USA; kdange@ncsu.edu; 4 Animal Health Department, Zoo Miami, Miami, FL 33177, USA; rodney.schnellbacher@miamidade.gov; 5 Conservation and Research Department, Zoo Miami, Miami, FL 33177, USA; Frank.Ridgley@miamidade.gov

**Keywords:** marine toad, passive integrated transponder (PIT), pathology, *Rhinella marina*, visible implant elastomer (VIE)

## Abstract

**Simple Summary:**

Passive integrated transponder (PIT) and visible implant elastomer (VIE) tags are commonly used to identify reptiles, amphibians, and fish. The aim of this study was to evaluate for pathologic changes associated with these tags in the marine toad (*Rhinella marina*). For the 56 toads in this study, all PIT tags were functional and 95% remained at the insertion site with little to no damage to the tissue. However, only 48% of VIE tags were functional, i.e., visible through the skin. Although there was little to no damage to the skin at the site of placement, the VIE was found to have migrated to the kidneys in 98% of toads as well as along the legs and sporadically in other internal organs. VIE in the kidney caused inflammation and damage to the vasculature that progressed in severity over time. Based on these findings, the authors recommend the use of PIT tags over VIE tags for identification of adult anurans, when feasible.

**Abstract:**

Passive integrated transponder (PIT) and visible implant elastomer (VIE) tags are commonly used to identify reptiles, amphibians, and fish. Despite reports of good retention rates and little to no negative effect on survival time, migration remains a concern and histologic changes have not been widely evaluated. Fifty-six wild-caught marine toads (*Rhinella marina*) were marked with a PIT tag in the left caudal thigh and a VIE tag over the right gastrocnemius muscle prior to transport to the North Carolina Zoo. Fourteen toads were then humanely euthanized on day 9, 15, 32, and 62 for postmortem examination and histopathology which were compared to 10 control toads with no tags. All PIT tags were functional and 95% remained at the insertion site with minimal to no histologic changes. Externally, 48% of VIE tags were visible through the skin at the original site of injection under fluorescent or UV light. Upon gross examination of the tissues, VIE tags had an overall retention rate of 62% at the injection site, with similar retention rates across time points. Migrated VIE material was visible grossly and histologically in the kidneys of 98% of toads and along the right leg, proximally and distally, in 25% of toads. VIE material was also found sporadically in mesentery, colon, and free in the coelomic cavity. Histologically, VIE material in the skin was surrounded by minimal to mild granulomatous inflammation while in the kidney it was associated with dilation of the small vessels, edema, and granulomatous nephritis that progressed in severity over time. Based on these findings, the authors recommend the use of PIT tags over VIE tags for identification of adult anurans, when feasible.

## 1. Introduction

Laboratory populations, zoological collections, and mark-recapture research often require identification of individual animals that are small in size, housed in large cohorts, and indistinguishable by external features. In amphibians, toe-clipping was initially a popular method for individual identification but has fallen out of favor due to risks of infection, effect on locomotion, and associated pain as well as the potential for misidentification due to digit regeneration [1,2]. Many types of tags have since been utilized to mark individuals for identification with two popular options being the passive integrated transponder (PIT) and visible implant elastomer (VIE) tags [3].

PIT tags are also referred to as “microchips” as they are small tubes containing an integrated circuit chip, capacitor, and antenna coil that sends an alphanumeric code to a reader when activated by the electro-magnetic field of the scanning device. PIT tags come in numerous sizes and are inserted under the skin or into the coelom surgically or using an implantation device. In adult anurans, effects on growth and survival appear to be minimal and retention rates are good despite the potential for tag expulsion [1,4]. However, PIT tags can be expensive and require a dedicated scanning device to be functional.

A more recent alternative is the VIE tag which was originally marketed for fish. VIE is a liquid elastomer that is commercially available in bright and fluorescent colors. The elastomer is mixed with a curing agent and injected “under the skin” (per manufacturer’s instructions) where it cures into a pliable solid over the course of a few hours at room temperature. The resulting tag is small, flexible, biocompatible, and can be visualized in daylight or enhanced with an ultraviolet (UV) light [5]. Along with fish, the use of VIE tags has been reported in amphibians, reptiles, and invertebrates with little effect on growth and survival but variable retention rates over time [2,6,7,8]. Within the literature across taxa, tag retention appears to be variably affected by species, individual size, injection location, volume injected, experience of the injector, and observer variability [3,6,7,9,10,11,12]. Tag migration, breakage, granuloma formation, and complete loss have all been reported [2,7,8,9,10,11,12,13,14,15,16]. Microscopic evaluation of VIE tagging sites has only been reported in shrimp (*Penaeus vannamei*) and stertlet (*Acipense ruthenus*) in which mild, chronic inflammation and fibrosis were found at the injection sites [14,16].

Despite significant concern for tag migration and loss in adult anurans, to the authors’ knowledge, histologic evaluation of PIT and VIE tagged anurans has not been reported [4,13]. The goal of this study was to evaluate gross and microscopic changes associated with PIT and VIE tags in anurans over time. Based on the aforementioned studies in other taxa and minimal reports of systemic effect, the authors hypothesized that there would be mild inflammation at the injection site and no associated pathology in the remaining organ systems.

## 2. Materials and Methods

Sixty-six marine toads (*Rhinella marina*) were wild-caught at Zoo Miami (Miami, FL, USA) as part of a routine invasive species population management program and enrolled in the current study. The work in this study was approved by the Zoo Miami Animal Care and Use Committee (#2020-6), the NC State University Institutional Animal Care and Use Committee (#20-270) and the North Carolina Zoo research review board. At the time of capture, 56 toads were randomly allocated to the ‘tagged’ group and 10 toads were left un-tagged as a control group. For the toads in the tagged group, at the time of capture in the field, the skin of the left caudal thigh was prepared with dilute betadine and a PIT tag injector (MK 25 PIT Tag Implanter, Biomark, Merck Animal Health, Boise, ID, USA) was used to place an 8 mm PIT tag (MiniHPT8 8 mm FDX-B Pre-loaded PIT tags, Biomark, Merck Animal Health, Boise, ID, USA) under the skin. The VIE (Visual Implant Elastomer Tags, Northwest Marine Technology, Inc., Anacortes, WA, USA) was prepared by thoroughly mixing the pink elastomer component with the clear curing agent in a 10:1 ratio [5]. The skin over the right gastrocnemius muscle was then prepared with dilute betadine and a tuberculin syringe was used to inject 0.02–0.04 mL of mixed elastomer under the skin until a visible 2–3 mm pink line of VIE was observed. The ambient temperature at the time of tag placement (evening, mid-August) was 26–30 °C and the mixed VIE was kept in a cooler on ice to prevent curing prior to injection. Tagged toads were then transported to the North Carolina Zoo (Asheboro, NC, USA). The day of capture, the control toads were euthanized via immersion in MS-222 (10 g/L, buffered with sodium bicarbonate). Toads were then preserved in neutral buffered 10% formalin and shipped to the North Carolina Zoo for pathologic evaluation and tissue evaluation.

On arrival, all toads were deemed healthy upon veterinary visual examination and PIT tag identifiers were confirmed with a handheld scanning device (601 Handheld Reader, Biomark, Merck Animal Health, Boise, ID, USA). At the North Carolina Zoo, the toads were housed in six separate tubs (178.8 × 81.28 × 35.56 cm) with access to hide boxes and a shallow pool of reconstituted reverse osmosis water. The tubs were cleaned daily and disinfected weekly. Animals were kept at ambient temperature, ranging from 20.5–27.7 °C, without supplemental heating or cooling. The light cycle was maintained according to working hours of animal husbandry staff (0800–1700 h). Each tub had a mesh screen covering affixed to the top to prevent escape and to partially block the artificial lighting. As part of a simultaneous nutrition study, all toads were fed a diet routinely used by the North Carolina Zoo consisting of gut-loaded adult brown house crickets (*Acheta domesticus*). The crickets were offered Hi Calcium Gut Loading Diet (Hi Calcium Gut Loading Diet, Mazuri®, St. Louis, MO, USA) and a small quantity of thinly sliced pieces of carrots and sweet potatoes prior to feeding to the toads. For half of the toads, the crickets were also dusted with Repashy Vitamin A Plus (Vitamin A Plus, Repashy Ventures, Inc., Oceanside, CA, USA) immediately before being provided to the toads.

Toads were then euthanized in randomly chosen groups of 14 individuals on day 9, 15, 32, and 62 to approximate 1 week, 2 weeks, 1 month, and 2 months. PIT tag identifiers were again confirmed, and functionality of the PIT tag was recorded. Toads were anesthetized with MS-222 (10 g/L, buffered with sodium bicarbonate) and euthanized via pithing. Following euthanasia, a full necropsy was performed by a board-certified pathologist (BT). External visibility of the VIE tag was considered positive if any amount of color was visible under the skin over the right gastrocnemius muscle under fluorescent or UV light. Location of any grossly visible internal VIE as well as the location of the PIT tag was recorded. Any gross abnormalities associated with either tag in any tissue, as well as any separate gross findings, were described.

All tissues except the heart and spleen, which were allocated to an unrelated study, were collected from all toads and placed in neutral buffered 10% formalin. Tissue from the PIT tag site, VIE tag site, lung, liver, kidney, gonads, gastrointestinal tract, and inguinal region was then routinely processed for histopathologic evaluation. VIE was recorded as present or absent in all tissues under microscopic examination and the amount of VIE in each tissue was subjectively scored on a scale from 0–4; 0: not present, 1: minimal, 2: mild, 3: moderate, 4: marked. Any inflammation or fibrosis associated with either tag was scored on the same scale. Small vessel distention with VIE was a common finding in the kidney and was consequently subjected to the same scoring system. All scoring was assigned by the same board-certified pathologist (BT) following best practice guidelines [17]. Descriptive statistics of scores for each tag and time point were calculated using standard software (Excel, Microsoft Corporation, Redmond, WA, USA). The Kruskal–Wallis test was used to compare amount of elastomer, inflammation score, fibrosis score, and renal vessel distention score across time points for each tag and location where applicable. The Mann–Whitney U test was used to compare the overall inflammation and fibrosis scores for PIT tags and VIE tags.

## 3. Results

### 3.1. Toad Demographics

Although the toads were collected randomly from Zoo Miami on a first seen basis, the control group of 10 untagged marine toads was half phenotypically male and half female. In the 56 tagged toads, 68% were male, 30% were female, and 2% were undetermined. Toad weights ranged from 50.0 g to 247.0 g (mean 144.2 g). The exact age of the wild-caught toads was unknown, but all were phenotypically sub-adult to adult.

### 3.2. Function

All PIT tags were functional at the time of euthanasia. Externally, 48% (27/56) of VIE tags were visible through the skin at the original site of injection under fluorescent or UV light.

### 3.3. Gross Results

**Figure 1:**
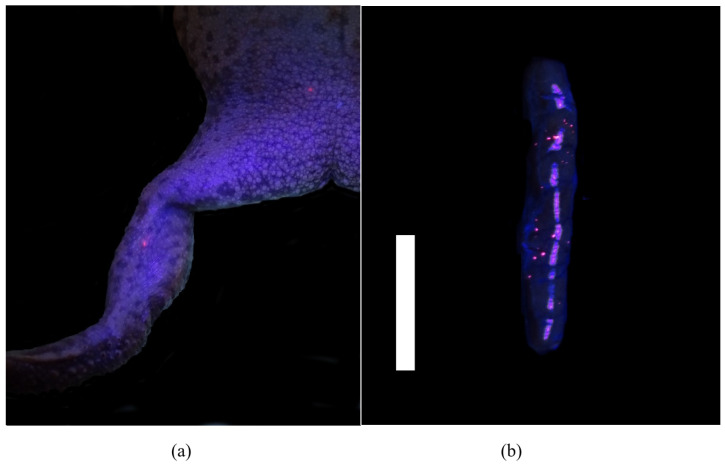
Gross postmortem examination of a marine toad (*Rhinella marina*) under UV light 9 days after visible implant elastomer (VIE) tag placement in the right distal limb. (**a**) Pinpoint fluorescent pink VIE is visible at the site of insertion under the skin over the right gastrocnemius muscle and migrated to the right inguinal region. (**b**) Multifocal pinpoint pink fluorescence throughout the parenchyma of the kidney, indicating systemic migration of VIE (white bar = 1 cm).

PIT tags were found grossly at the site of insertion in 95% (53/56) of toads with migration noted in three (5%) toads. In one toad euthanized at the 32-day time point, the PIT tag had migrated distally along the left leg to the metatarsus. At the 62-day time point, two PIT tags had migrated proximally, one to the left inguinal region (Figure 1) and one was found lateral to the lumbar spine on the left side in the subcutaneous tissue.

While trimming, VIE was grossly visible in the skin or muscle of the right distal limb under fluorescent or UV light in 62% (35/56) of toads. Locally migrated VIE was grossly visible proximal or distal to the site of insertion in the right leg in 14 toads (25%). VIE was also visible grossly in the kidneys of 64% (36/56) of toads. In the kidney, VIE appeared as pinpoint bright and fluorescent pink foci scattered throughout the parenchyma (Figure 1). Of those animals, 33% had grossly visible VIE only in the right kidney, 6% had grossly visible VIE only in the left kidney, and 61% had grossly visible VIE in both kidneys. Further VIE was grossly visible in the peritoneum (5/56), free in the coelomic cavity (3/56), in the facial planes of the left leg (2/56), and in the lamina propria of the colon (1/56). No VIE was grossly visible in all other organs assessed including the lungs, liver, and urogenital system.

### 3.4. Microscopic Results

Control toads with no tags had moderate mononuclear inflammation or granuloma formation associated with parasites in the lungs, skin and muscle. No inflammation was found in the kidneys of any control toads.

PIT tag sites in the left caudal thigh were characterized by a large vacant space within either the muscle fascicles or adjacent epimysium where the tag was present prior to processing, sometimes surrounded by a thin band of fibrosis with occasional mononuclear cellular infiltrate. In the majority of toads (61%; 34/56) there was no inflammatory reaction present. When noted, granulomatous inflammation associated with the PIT tag was considered minimal in 27% (15/56), mild in 11% (6/56), and moderate in 2% (1/56) of toads with no toads having marked inflammation. The majority of toads (66%; 37/56) also had no fibrosis associated with the PIT tags while 21% (12/56) of toads had minimal fibrosis and 13% (7/56) had mild fibrosis. On average across time points, PIT tags were associated with minimal to no granulomatous inflammation or fibrosis and no significant change over time (Table 1). There was also no significant difference in inflammation scoring between toads with PIT tags and control toads, in which inflammation was associated with encysted parasites.

VIE was identified in the tissues of the right distal limb of 77% (43/56) of toads. VIE appeared as dark granules within the dermis (7/56), subcutis (11/56), epimyseum (8/56), muscle (7/56), or in multiple layers simultaneously (10/56) and fluoresced pink under UV light. Scoring of the original injection site over the right gastrocnemius muscle revealed, on average, a minimal to mild amount of VIE at all time points with no significant difference found between time points. In 48% (27/56) of toads, VIE at the site of injection in the right distal limb was associated with infiltration of macrophages and occasional eosinophils, regardless of the tissue layer in which it was found. Rare multinucleated giant cells and intrahistiocytic polymer were also frequently observed, indicating foreign body reaction. Granulomatous inflammation scores were minimal in 20% (11/56), mild in 23% (13/56), and moderate in 5% (3/56) of toads with no toads having marked inflammation at the site of injection. Fibrosis was associated with VIE tags in 36% (20/56) of toads with the majority having minimal fibrosis (29%; 16/56) and few having mild fibrosis (7%; 4/56). On average across time points, VIE tags were associated with minimal to no granulomatous inflammation or fibrosis at the site of injection (Table 1). No significant difference was found in inflammation or fibrosis scores between time points. No significant difference was also found in inflammation or fibrosis score at the injection site when comparing VIE tags to PIT tags at any time point or when comparing overall scores.

**Figure 2:**
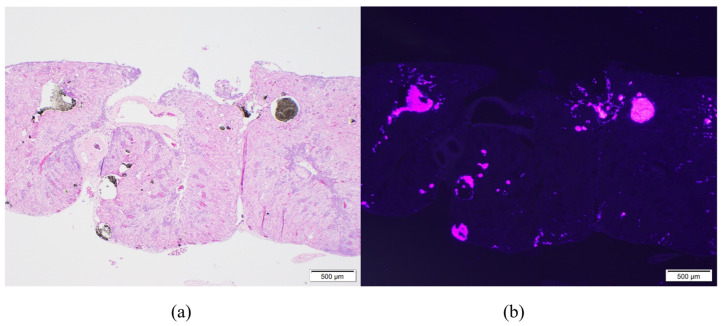
Microscopic examination of the kidney of a marine toad (*Rhinella marina*) 62 days after visible implant elastomer (VIE) tagging in the right distal limb. 10× magnification shows migrated VIE material in the small vasculature (vasa recta) of the kidney causing distention of the vessels. (**a**) On standard light microscopy, VIE is characterized as dark granules while under UV light (**b**) the VIE material fluoresces pink.

**Figure 3:**
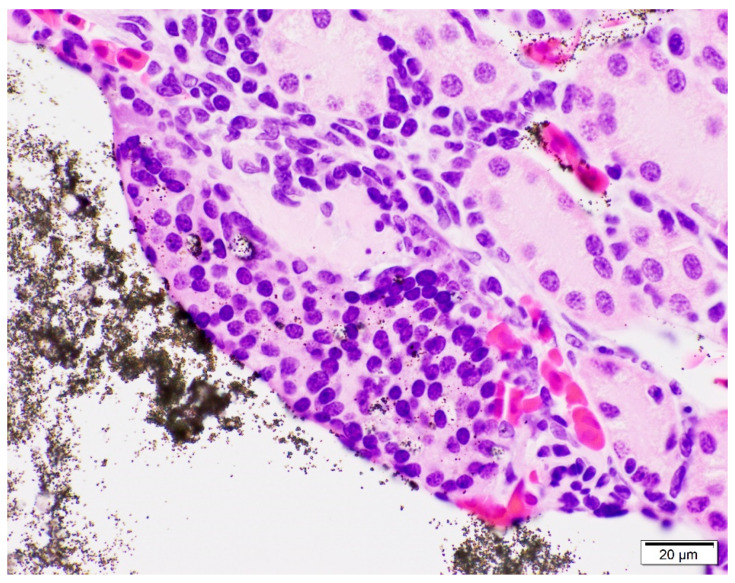
Microscopic examination of the kidney of a marine toad (*Rhinella marina*) 62 days after visible implant elastomer (VIE) tagging in the right distal limb. 100× magnification shows granulomatous nephritis associated with migrated VIE material in the small vasculature (vasa recta) of the kidney. Phagocytosis of VIE granules, visible within the surrounding macrophages, provides further evidence for the inflammatory reaction to the VIE material in the kidney.

On microscopic examination of the kidneys, VIE was found in 98% (55/56) of toads. VIE was again characterized as dark granules that were fluorescent pink under UV light, primarily found within small vasculature including subcapsular vessels and vasa recta (Figure 2). Phagocytosed VIE granules were also visible in adjacent macrophages (Figure 3). The average amount of VIE in the kidney was minimal to mild for the 9-day time point, but mild to moderate for all subsequent time points. VIE material in the small renal vessels was associated with secondary lesions of small vessel distention, adjacent interstitial edema, and granuloma formation which increased over the time of the study. Secondary small vessel distention from renal VIE material was found in 80% (45/56) of toads and was characterized as minimal in 44% (25/56), mild in 34% (19/56), and moderate in 2% (1/56) of toads. Although no significant difference was found across time points, distention was generally minimal in the early time points but minimal to mild at the 32- and 62-day time points. VIE within the small vessels of the kidney was also associated with granuloma formation in 23% (13/56) of toads, characterized by infiltration of macrophages and phagocytosis of VIE granules. The number of toads identified with granulomatous nephritis increased from 4% (2/56) in the 9- and 15-day time points, to 7% (4/56) and 9% (5/56) in the 32- and 62-day time points, respectively.

Granulomatous inflammation was also associated with the focal VIE material observed within the lamina propria of the colon of one toad at day 15 and in the mesentery of one toad at day 62. Lesions associated with PIT or VIE were not observed in any examined section of lung, liver, gonads, inguinal region, and all remaining gastrointestinal tracts. As with control toads, dermal granulomas associated with parasites and pulmonary nematodes were commonly observed.

## 4. Discussion

In the marine toad, PIT tags placed subcutaneously in the caudal left thigh were associated with minimal to no granulomatous inflammation at the site of insertion and nominal migration up to two months following placement. VIE tags placed subcutaneously in the right distal limb of marine toads were also associated with minimal to no granulomatous inflammation in the skin but showed extensive migration throughout the body. Most notably, VIE consistently migrated to the kidneys, often as early as one week after placement, where it was associated with small vessel distention, rupture, and granulomatous nephritis which progressed over time. To the authors’ knowledge, this is the first report of renal migration of VIE in any species.

Histologic findings of VIE in the vasculature and the pattern of migration are consistent with uptake into the renal-portal system, which carries blood from the caudal half of the body (i.e., the hind limbs) through the kidney before entering the post-caval vein [18]. The location of VIE in the small vessels and vasa recta suggests migration through the renal portal vasculature, into the capillary network, where it appeared to become lodged. Accumulation of VIE in the vasa recta led to progressive distention and rupture of the vessels, secondary granuloma formation, and engulfment of the VIE material by macrophages. It is unclear if the migration seen here is unique to the population or species in this study, as published reports of full postmortem and microscopic examination of VIE tagged animals are limited to the two aforementioned studies in shrimp and stertlet, which only report changes at the injection sites [14,16]. No previous report of histopathologic evaluation of VIE tagged anurans was found in the published literature.

There is a vast expanse of literature on VIE tag retention by species, in which many studies in other taxa report good retention rates as determined by external visibility [1,2,3,19,20]. Studies in amphibians suggest good retention in caecilians and variable retention depending on the species of salamander evaluated [10,11,21,22]. However poor retention of VIE tags has been reported in adult anurans and it is possible that anatomic or physiologic factors specific to anurans increase the rate of tag migration in these species [13]. Individual size and sex have also been suggested as contributing factors to tag loss and mortality in small fish and salamanders but the skewed morphometrics of this population (body weight 50–247 g; 68% male, 30% female, and 2% undetermined), precluded statistical analysis [11,12,19,23].

The marine toads in this study had a high parasite load with widespread metacercaria-associated granulomatous inflammation that was often more significant in the dermis than that associated with the VIE or PIT tags. Parasite burdens are to be expected in certain populations, especially in mark-recapture studies of wild animals, and inflammation due to tagging was interpreted relative to this routine finding. Importantly, renal pathology was associated solely with VIE. The concurrent nutritional study evaluating the effects of dietary vitamin A supplementation was unlikely to have contributed to inflammation or migration of VIE in this population due to the anti-inflammatory properties of vitamin A in other taxa [24].

Variables associated with VIE preparation and placement may have played a role in VIE retention at the site of injection. Subjectively, intramuscular VIE had a higher retention rate but also had decreased visibility externally through the skin and more inflammatory infiltrate than subcutaneous VIE. This finding was also suggested in the histologic study of VIE tagged stertlet [16]. Unfortunately, a statistical analysis was not possible in the current population due to the low number of individuals with VIE in a single tissue layer alone. Large vessels including the femoral and sciatic veins are present along the flexor surface of the stifle, just proximal to the location of VIE injection on the caudal aspect of the right distal limb. It is possible that VIE was injected directly into the vasculature or in close enough proximity that this contributed to vascular migration. However, a study in adult Kihansi spray toads (*Nectophrynoides asperginis*) showed marked gross migration despite VIE placement in the proximal or distal half of the forelimb or hindlimb [13]. In a study in smooth newts (*Lissotriton vulgaris*), higher rates of migration were found when VIE tags were placed in the forelimbs compared to the hindlimbs [11]. These findings suggest that migration may occur regardless of the location of VIE placement.

The volume of VIE injected ranged from 0.02–0.04 mL. This was less than the manufacturer’s recommendation of 0.1 mL minimum that can be reasonably handled and mixed [5]. The volume was based on the small size of the injection site in the marine toads and the desired size of the VIE tags. A previous study in larval salamanders found that smaller marks had higher retention rates and it is possible that larger volumes of VIE stimulate a more robust inflammatory or foreign body reaction contributing to tag breakage and migration [23]. Alternatively, a larger injected volume may force VIE into deeper tissues or local vessels contributing to reduced visibility, inflammation, and migration. It is also unclear if the migrated VIE material is from a cured tag that has broken down or if the curing process was unsuccessful. Operator error, product malfunction, or low ambient temperatures may have contributed to inadequate curing, allowing for migration of VIE. However, a study in larval Pacific lamprey (*Lampetra tridentata*) found that uncured VIE had equally long retention rates as cured VIE and a recent study in zebrafish (*Danio rerio*) found that uncured tags had a higher retention rate, suggesting that curing may not be required for tag retention [15,20].

Identifying the primary factor associated with VIE migration was out of the scope of this project but results of this study suggest that the main cause for poor VIE tag retention in adult anurans may be migration through the vasculature as well as along the fascial planes of the leg. The effects of progressive granulomatous nephritis and fibrosis associated with vascular VIE migration into the kidney is unknown. A lack of increased mortality rates with VIE tagging across the available literature suggests that the effects are likely subclinical. However, studies assessing markers of renal function over time would be necessary to elucidate the true effects of VIE tagging on the kidneys. Furthermore, external evidence of tag breakage and migration or loss is widely reported in other species and taxa which may suggest that similar systemic migration is occurring. Future studies should be considered to evaluate the internal migration of VIE and assess for any short or long-term medical and welfare implications.

## 5. Conclusions

In the marine toad, PIT tags were found to have good retention at the site of implantation with minimal to no inflammation or other associated histologic changes up to two months after placement. The VIE tags also had minimal to no inflammation at the site of injection but function was compromised by loss from the original site and a high rate of systemic migration. VIE appears to spread along fascial planes as well as through vasculature where it is consistently carried to the small vessels of the kidneys. Within the kidneys, VIE material was associated with progressive granulomatous nephritis with unknown long-term implications. The authors advise caution when using VIE tags, especially if longevity is expected or if the renal system is to be studied concurrently. If possible, the authors recommend the use of PIT tags over VIE tags for individual identification of anurans.

## Figures and Tables

**Table 1:** Average numerical score for tag-associated histopathologic findings of marine toads (*Rhinella marina*) euthanized at four time points following PIT tag placement in the left caudal thigh and VIE tag placement in the right distal limb. Findings included the amount of VIE in each tissue, granulomatous inflammation, fibrosis, and small vessel distention, and each was assigned a score from 0–4; 0: not present, 1: minimal, 2: mild, 3: moderate, 4: marked. For each finding, the average score for all 56 tagged toads is reported in the overall column.

	Control	Day 9	Day 15	Day 32	Day 62	Overall
	*n* = 10	*n* = 14	*n* = 14	*n* = 14	*n* = 14	*n* = 56
**PIT ^a^ tag: skin**						
Granulomatous inflammation	0.50	0.43	0.50	0.42	0.78	0.52
Fibrosis	0	0.57	0.64	0.43	0.21	0.46
**VIE ^b^ tag: skin**						
Elastomer amount	-	1.90	1.57	1.14	1.50	1.53
Granulomatous inflammation	0	0.86	0.57	0.79	1.07	0.82
Fibrosis	0	0.57	0.64	0.36	0.14	0.43
**VIE ^b^ tag: kidney**						
Elastomer amount	-	1.80	2.28	2.64	2.57	2.30
Small vessel distention	0	1.00	1.00	1.14	1.57	1.18

^a^ MiniHPT8 8 mm FDX-B Pre-loaded PIT tags, Biomark, Merck Animal Health, Boise, ID, USA. ^b^ Visual Implant Elastomer Tags, Northwest Marine Technology, Inc., Anacortes, WA, USA.

## Data Availability

Data available on request.
